# A Conformation Variant of p53 Combined with Machine Learning Identifies Alzheimer Disease in Preclinical and Prodromal Stages

**DOI:** 10.3390/jpm11010014

**Published:** 2020-12-26

**Authors:** Giulia Abate, Marika Vezzoli, Letizia Polito, Antonio Guaita, Diego Albani, Moira Marizzoni, Emirena Garrafa, Alessandra Marengoni, Gianluigi Forloni, Giovanni B. Frisoni, Jeffrey L. Cummings, Maurizio Memo, Daniela Uberti

**Affiliations:** 1 Department of Molecular and Translational Medicine, University of Brescia, 25123 Brescia, Italy; giulia.abate@unibs.it (G.A.); marika.vezzoli@unibs.it (M.V.); emirena.garrafa@unibs.it (E.G.); maurizio.memo@unibs.it (M.M.); 2 GolgiCenci Foundation, 20081 Abbiategrasso, Italy; letizia.polito@gmail.com (L.P.); a.guaita@golgicenci.it (A.G.); 3 Department of Neuroscience, IRCCS-Istituto di Ricerche Farmacologiche “Mario Negri”, 20156 Milan, Italy; diego.albani@marionegri.it (D.A.); gianluigi.forloni@marionegri.it (G.F.); 4 Laboratory of Alzheimer’s Neuroimaging and Epidemiology (LANE), IRCCS Istituto Centro San Giovanni di Dio Fatebenefratelli, 25125 Brescia, Italy; mmarizzoni@fatebenefratelli.eu; 5 Department of Clinical and Experimental Sciences, University of Brescia, Lombardy, 25123 Brescia, Italy; alessandra.marengoni@unibs.it; 6 Memory Clinic, University Hospitals and University of Geneva, 1205 Geneva, Switzerland; giovanni.frisoni@hcuge.ch; 7 Department of Brain Health, School of Integrated Health Sciences, University of Nevada Las Vegas (UNLV) and Cleveland Clinic Lou Ruvo Center for Brain Health, Las Vegas, NV 89106, USA; jcummings@cnsinnovations.com; 8 Molecular Markers Laboratory, IRCCS Istituto Centro San Giovanni di Dio Fatebenefratelli, 25125 Brescia, Italy

**Keywords:** blood-based biomarker, Alzheimer’s disease, machine learning, β-amyloid, conformation variant of p53

## Abstract

Early diagnosis of Alzheimer’s disease (AD) is a crucial starting point in disease management. Blood-based biomarkers could represent a considerable advantage in providing AD-risk information in primary care settings. Here, we report new data for a relatively unknown blood-based biomarker that holds promise for AD diagnosis. We evaluate a p53-misfolding conformation recognized by the antibody 2D3A8, also named Unfolded p53 (U-p53^2D3A8+^), in 375 plasma samples derived from InveCe.Ab and PharmaCog/E-ADNI longitudinal studies. A machine learning approach is used to combine U-p53^2D3A8+^ plasma levels with Mini-Mental State Examination (MMSE) and apolipoprotein E epsilon-4 (APOEε4) and is able to predict AD likelihood risk in InveCe.Ab with an overall 86.67% agreement with clinical diagnosis. These algorithms also accurately classify (AUC = 0.92) Aβ^+^—amnestic Mild Cognitive Impairment (aMCI) patients who will develop AD in PharmaCog/E-ADNI, where subjects were stratified according to Cerebrospinal fluid (CSF) AD markers (Aβ42 and p-Tau). Results support U-p53^2D3A8+^ plasma level as a promising additional candidate blood-based biomarker for AD.

## 1. Introduction

Alzheimer’s disease (AD) is a progressive and devastating neurodegenerative disease affecting nearly 50 million elderly worldwide [1]. This clinical syndrome is characterized by a progressive deterioration in cognitive and functional abilities, leading to impaired everyday activities and eventual death. Delayed diagnosis, the lack of efficacious therapies, and the associated chronic disability render the disease a calamity [2]. Although significant efforts have been directed toward the identification of potential pharmacological targets, no disease-modifying therapy has yet been approved. Among the contributing reasons for these failures is the absence of biomarkers for the early detection and the biological stratification of the participants in clinical studies [2]. Biomarkers are also needed to identify high-risk individuals before cognitive symptoms manifest, allowing early interventions before the brain is irreversibly damaged.

It is now well established that clinical manifestations of AD are preceded by a long preclinical stage, during which pathophysiological processes might occur not only in the brain [3,4] but also at the periphery [5,6]. During this period, therapies might be most effective to prevent, slow, or even stop the disease. 

One of the well-recognized upstream events in AD pathogenesis is amyloid β protein (Aß) deposition in the brain; although it is insufficient to cause cognitive deterioration, it may be sufficient to induce downstream pathological changes that ultimately lead to cognitive deterioration [7]. A widely held view is that amyloid biomarkers represent the earliest evidence of AD neuropathological changes currently detectable in living persons [7]. Cerebrospinal fluid (CSF) Aß levels and amyloid PET scanning are validated early biomarkers of AD, but the invasive nature of the former and the high cost of the latter limit their application. Blood-based biomarkers represent promising solutions for AD diagnosis and research since they could provide a non-invasive test to be used in the first steps of the diagnostic workflow, ruling out those subjects who do not have underlying AD pathophysiology from further analyses [8,9]. In the discovery of such biomarkers, one strategy has been to seek in blood what is already known in the brain. Although for many years identifying meaningful Aß biomarkers in blood has been a challenge, recently, highly sensitive and precise assays for its measurement in plasma, strongly reflecting brain amyloidosis, have emerged [10]. However, some individuals with normal CSF-Aß or negative amyloid PET progress to AD, strongly suggesting the need to explore alternative and complementary biomarkers. 

We previously demonstrated that p53 conformational alterations were found in different cell types derived from AD patients (fibroblasts, immortalized B lymphocytes, and peripheral blood mononuclear cells (PBMCs)) as well as in in vitro and in vivo models [11,12], using several experimental approaches. More recently, we demonstrated that, upon different redox stressors exposure, several misfolding p53 conformations can exist [13] with different affinity for different anti-p53 antibodies. Here, we propose the detection in plasma of a misfolding p53 conformational variant recognized by the novel conformational antibody 2D3A8 (U-p53^2D3A8+^) as a predictive biomarker of AD risk. Although p53 has been seen for more than two decades as implicated in the cell death occurring in neurodegeneration, a growing body of evidence supports the notion that p53-induced adaptive responses, including immune response control, redox balance, and neurite rearrangement, are instead gradually dysregulated during the AD *continuum* [14,15,16,17,18,19,20,21]. Recently, several research studies have described the pleiotropic role of functional/unfunctional p53 in the brain and its possible role in AD pathogenesis, describing how dysregulated p53 signaling and the loss of its canonical biological function may exacerbate AD pathology [22].

The expressions of APP and ß-secretase (BACE1) are negatively regulated by p53 [23], corroborating the contribution of p53 to preventing APP pro-amyloidogenic process. The link between γ-secretase members and p53 is more complex. p53 is regulated by and regulates members of the γ-secretase complex. In particular, p53 increases the expression of presenilin enhancer 2 (Pen-2), which, in turn, negatively regulates p53 expression. Presenilin 1 is also a negative regulator of p53, whereas presenilin 2 increases p53 transcriptional activity. Together, these findings highlight p53 as a tight regulator of APP processing [24]. 

In the present work, for the first time, we tested and validated the antibody 2D3A8 for the detection of a p53-misfolding conformation in plasma samples derived from two different longitudinal cohorts where prodromal and preclinical AD were included. Using a machine learning approach that combines U-p53^2D3A8+^ plasma levels with clinical variables, we developed an algorithm able to stratify AD risk. 

## 2. Materials and Methods

### 2.1. Participants and Clinical Phenotyping

Participants were aged between 60 to 80 years old. We analyzed 375 blood samples derived from single-center Italian studies and a multicenter cohort in Europe. These included 264 plasma samples selected from InveCe.Ab, a 4 year longitudinal study in a population of community-dwelling elderly inhabitants of Abbiategrasso, Italy [25], and 111 plasma samples from PharmaCog/E-ADNI, a European multicenter memory-clinic-based study involving amnestic Mild Cognitive Impairment patients (aMCI) [26]. 

Cohort’s characteristics are summarized in Table 1, and the design of the entire InveCe.Ab study is reported in Table S2. 

In detail, in InveCe.Ab, frozen EDTA-Plasma samples derived from 64 cognitively healthy subjects (CN) and 26 MCI were available from three sampling periods 2 years apart (Baseline, T_1_ and T_2_) during a 4-year follow-up [25] (Tables S3 and S4). AD was diagnosed using the National Institute of Neurological Disease and Communicative Disorders and Stroke–Alzheimer’s Disease and Related Disorders Association (NINCDS/ADRDA) criteria [27] and based on the diagnostic workup according to the European Federation of Neurological Societies guidelines [28]. In PharmaCog/E-ADNI, thirteen clinical centers consecutively recruited 147 aMCI patients, of whom 111 were included in this study. The neuropsychological battery test and inclusion and exclusion criteria have been described elsewhere [26]. Baseline CSF and blood were preprocessed, frozen, and stored at each site according to a standardized protocol and in line with the Alzheimer’s Association quality control program [29]. Clinical diagnosis of neurodegenerative disorders was made according to the conventional criteria [27,30,31]. For CSF biomarker, we used published cutoffs (Aβ42 < 550 pg/mL, t-Tau > 225.6 pg/mL, and p-Tau > 52 pg/mL) [26,32,33]. 

The two cohorts were chosen according to the context of use (COU) to investigate and the outcomes for which the single cohort was designed (conversion to AD, Aβ status). For the InveCe.Ab study, the outcome was the evaluation of U-p53^2D3A8+^ in relation to AD-conversion, both in non-demented subjects and in subjects with mild cognitive symptoms (MCI), classified according to clinical international criteria [27]. For PharmaCog/E-ADNI, the outcomes were the correlation of U-p53^2D3A8+^ with: (i) AD-associated brain pathological changes (i.e., CSF AD biomarkers) and (ii) the rate of MCI-to-AD conversion according to their Aβ status. 

In addition, 114 plasma samples from the population-based study ANZIANI IN-RETE [34], a randomized sample of the elderly population of Brescia, Italy, affected by different chronic diseases and multi-morbidities, were also tested to evaluate U-p532^D3A8+^ distribution in age-related co-morbidities. More information in Supplementary Materials. 

All the studies were approved by the appropriate institutional ethics committee, and all the participants gave written informed consent. InveCe.Ab: Clinical trials.gov, NCT01345110; ANZIANI IN-RETE: reference number 0144116; Pharmacog/E-ADNI: parere 36/2010.

### 2.2. Sample Size Considerations for InveCe.Ab 

The power calculation and sample size estimation were performed in accordance with the AD conversion rate within the InveCe.Ab study: among 1039 CN at baseline, 10 converted to AD (0.96%), and among 101 MCI, 10 converted to AD (9.9%) within 4 years. The effect size was estimated based on the observation that at *T*_2_, 692 were stable CN and 40 were MCI. The multiple regression power calculation of sample size considered 6 independent variables (*Sex, Education level, Age, APOEε4, MMSE, plasma_U-p53^2D3A8+^*), the effect size of 0.666 for stable CN and 0.5882 for stable MCI, a sensitivity > 80% and a theoretical sensitivity of 50% (statistical power of 0.8 and a significance level of 0.05). Therefore, using this analysis in addition to the 20 converters of the longitudinal study, the number of stable CN and MCI would have been not less than 20.4 (≅21) and 23.09 (≅23) subjects, respectively. The different subgroups were then selected according to age, gender, comorbidities index, severity index, and clinical category matched.

### 2.3. Immunoassay for Plasma U-p53^2D3A8+^ Biomarker Analysis

An indirect in-house ELISA with 2D3A8 antibody (epitope aa 282-297) was used to measure U-p53 in plasma. The 2D3A8 anti-p53 antibody is under patent fully owned by Diadem srl (PCT/EP2015/072094/family 15122V), and more details about the antibody are reported in Table S1. More information related to the specificity of 2D3A8 for a conformational variant of p53 and assay reproducibility are reported in Figures S1–S3.

Briefly, 100 μL of diluted plasma and reference-control peptides at different concentrations were coated on a plate overnight at 4 °C. Results were extrapolated from the peptide standard curve ranging from 1.87–30 ng. Thus, considering that the evaluation of U-p53 detected by the assay is expressed in relation to a short reference peptide, results have been expressed in arbitrary units (a.u.) not to confuse the measure with the estimation of p53 protein concentration in human plasma. 

### 2.4. Statistical Analysis

Linear Mixed Effects (LME) [35] regression was used to model U-p53^2D3A8+^ levels across time, as well as across diagnostic status. We ran two separate models: one comparing stable CN with CN converting to AD, and the other stable MCI with MCI converting to AD. The description of the model output is described in Table S6. 

A non-parametric method Regression Tree (RT) [36,37], a type of Machine Learning technique [38,39], was used to deliver an Early Warning System that strongly predicts risk zones in terms of AD prevalence or Aβ status (ranging from 0—no risk—to 1—maximum risk). When the dependent variable (Y) is dichotomous, the prediction in each terminal node is the relative frequencies of 1 in the node itself. The estimation process is based on the ten-fold cross-validation, through which the data are partitioned into *v* nearly equally sized folds, and next running *v* interactions of training and validation to such an extent that within each interaction, a different fold of the data is held out for validation, while the remaining (*v* − 1) folds are used for learning. Since InveCe.Ab dataset showed a longitudinal structure, we took advantage of this by using the rolling window procedure [40], proposed in finance for time series, in order to predict the diagnosis of each patient, where Sex, Education Level, Age, MMSE, APOEε4 and plasma_U-p53^2D3A8+^ are the covariates. 

**Figure 1:**
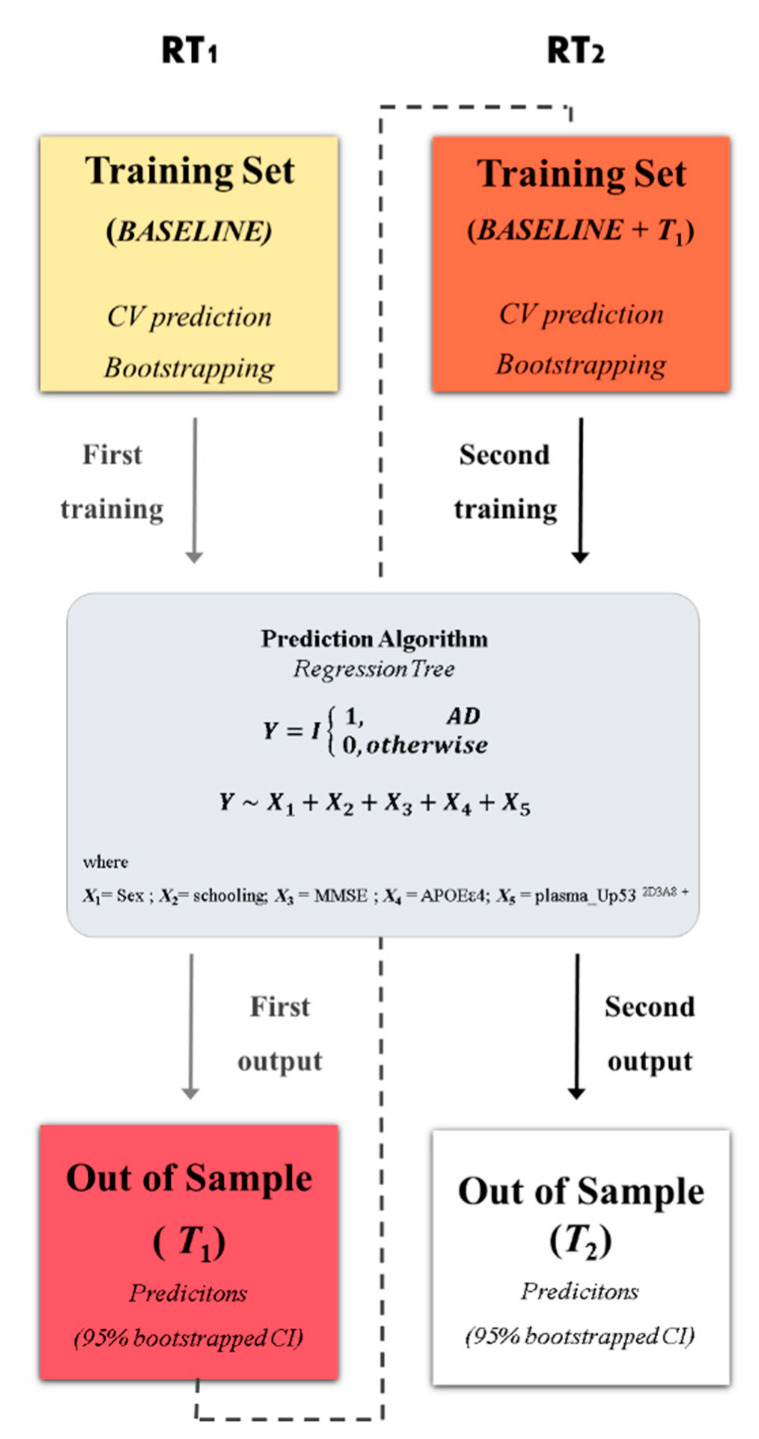
Statistical framework. The procedure for obtaining the optimal decision tree with a rolling window procedure. In the first step, a regression tree (RT1) algorithm was trained on InveCe.Ab data at baseline (first training set represented by a yellow box). The model was grown using five covariates (X1, …, X5, listed in the grey box), which predict the dichotomized diagnosis (outcome: non-Alzheimer’s disease (AD) = 0; AD = 1). The output obtained was then tested on T1 (first window, red box), an independent dataset collected on the same patients after two years. In the second step, the training set was reinforced by joining baseline and T1 (second window, orange box); the same model in the grey box was calibrated on it (RT2) and tested on T2 (second window, white box). RT predictions were bootstrapped, obtaining a 95% CI. Additional information on the procedure is provided in Table S7 and Figure S5.

In detail, the prognostic accuracy of the proposed RT model used in InveCe.Ab is obtained because the RT is calibrated within a starting window (Baseline—first training set); then, such model is used to forecast the Alzheimer conversion of patients after two years (T_1_, first out of sample). Since, in the original dataset, AD patients are not available at the baseline, a small-scale training set [41] has been included. The procedure is repeated, joining Baseline and T_1_ (second training set) for the calibration of the model and testing Alzheimer conversion after four years (T_2_, second out of samples). A schematic representation is reported in Figure 1 and additional information in Table S7 and Figure S4. 

In order to evaluate the performances of methods introduced in this study, Receiver Operating Characteristic (ROC) analysis is used, extracting additional metrics such as Area Under the Curve (AUC), sensitivity, and specificity. Corresponding 95% confidence interval (CI), computed with 10,000 stratified bootstrap replicates, are reported. Results obtained are then compared with a well-known parametric model such as Logit, extracting predictions and computing the same metrics proposed in decision trees. Moreover, rank correlations [42], test on proportion, Fisher exact test, Wilcoxon rank sum test (U of Mann-Whitney), Kruskal–Wallis test, ANOVA test, De-Long test were applied. Analyses were performed using R, version 3.4.4.

## 3. Results

We tested 264 plasma samples derived from InveCe.Ab [25] with the anti-p53 antibody 2D3A8 to evaluate the expression of a misfolding conformation variant of p53 (U-p53^2D3A8+^) during disease progression (Table 1). According to the sample size power calculation that considered the percentage of AD conversion, a total of 64 CN (10 progressed to AD and 17 to MCI) and 26 MCI (9 progressed to AD) were evaluated. Time-series data for U-p53^2D3A8+^ were collected at three time points every two years (Baseline, T_1_, and T_2_) using an in-house immunoassay. Five different groups were considered according to diagnosis at T_2_: Stable-CN, CN converted to MCI (CN-to-MCI) or AD (CN-to-AD), Stable-MCI, and MCI converted to AD (MCI-to-AD). At baseline, the CN-to-AD converters group showed a statistically significant elevation of U-p53^2D3A8+^ levels compared with stable-CN, which was more evident at T_1_ (*p* < 0.05) and T_2_ (*p* < 0.0001). Differences in U-p53^2D3A8+^ were also observed at T_2_ in MCI-to-AD converters compared to Stable-MCI (*p* < 0.01). An increase in U-p53^2D3A8+^ plasma levels was found in CN-to-MCI progression (*p* < 0.0001), linking U-p53^2D3A8+^ with the worsening of cognitive decline (Table S5).

**Figure 2:**
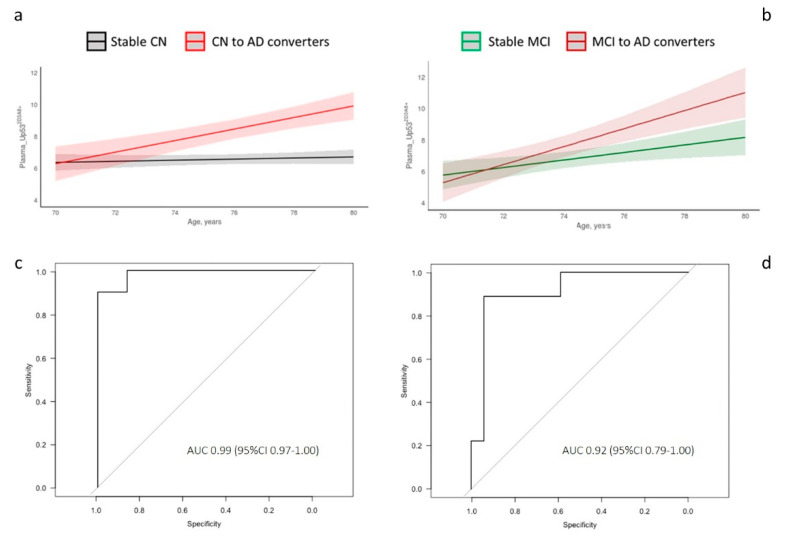
Longitudinal analysis and diagnostic accuracy of clinical Alzheimer’s disease of plasma_U-p53^2D3A8+^ in InveCe.Ab. Linear mixed-effects (LME) was used to model U-p53^2D3A8+^ levels across time and across disease progression in InveCe.Ab (**a**,**b**). ROC curves performed at T_2_ after the conversion to decipher the accuracy of AD diagnosis (**c**,**d**). U-p53^2D3A8+^-discriminated Stable CN vs. CN-to-AD with specificity of 1.0 and sensitivity of 1.0 (**c**). U-p53^2D3A8+^-distinguished Stable MCI vs. MCI-to-AD with 0.94 specificity and 0.89 sensitivity (**d**). AUC, area under the curve. Figure S4 reports box and whiskers representation of U-p53^2D3A8+^ across follow-up (baseline, T_1_, and T_2_) and disease stratification (CN-Stable, CN to MCI, and CN to AD on the left and MCI Stable and MCI to AD on the right). Results were extrapolated from a short-reference peptide standard curve and are expressed in arbitrary units (a.u.).

LME longitudinal analysis showed that the trajectory slope of CN-to-AD converters was steeper compared to stable CN (Figure 2a), whereas those of Stable MCI and MCI-to-AD showed a tendency to overlap (Figure 2b), corroborating that U-p53^2D3A8+^ plasma levels correlate with the continuum of the disease. ROC curves performed at T_2_ (after the conversion) displayed an AUC of 0.99 (sensitivity and specificity = 100%) and an AUC of 0.92 (sensitivity = 88.9% and specificity = 94.1%) in discriminating Stable-CN vs. CN-to-AD and Stable-MCI vs. MCI-to-AD, respectively (Figure 2c,d). 

**Figure 3:**
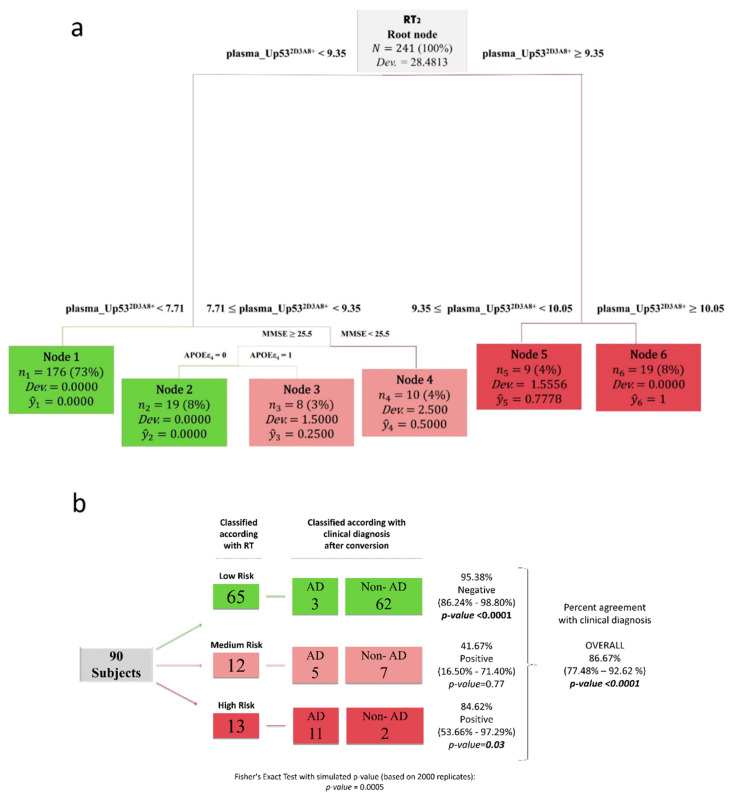
U-p53^2D3A8+^ plasma-based machine learning approach for AD-likelihood risk. Regression trees (RTs) combined with the rolling window procedure were produced using sex, education, age, MMSE, APOEε4, and plasma_U-p53^2D3A8+^ as covariates to predict AD-likelihood risk. (**a**) The tree structure obtained by RT_2_, which identified 6 nodes, representing 3 AD risk categories: low- (in green), middle- (in pink), and high-risk (in red). (**b**) Schematic representation of the at-AD-risk stratification, obtained using fresh data at T_2_ from the InveCe.Ab dataset (second window of our procedure) in RT_2_. This blinded test set (87 observations) was classified and stratified according to AD risk established by RT_2_. The three missing subjects at T_2_ (3 MCI converted to AD) were recovered from the first output prediction at T_1_. On the left, we report the RT classification; on the right, the clinical diagnosis after conversion. Test on the proportion evaluated whether the proportion of subjects classified as non-AD/AD was significantly different from 0.5, and Fisher’s exact test was used to estimate the association between the clinical diagnosis and the risk stratification induced by the RT. ŷ, the relative frequency of patients, clustered within the same final node; Dev., deviance. Cutoffs are expressed in arbitrary units (a.u.) and related only to the here-proposed in-house ELISA with a short-reference peptide used as the internal standard.

To test the performance of plasma U-p53^2D3A8+^ in predicting AD-likelihood risk, we used an integrated regression tree (RT) algorithm, a machine learning technique, with the rolling window procedure to construct a two-step procedure that provides an early warning system strongly predicting risk zones of AD prevalence. The tree structure obtained by RT_2_ (Figure 3a) selected U-p53^2D3A8+^ plasma levels as the most important differentiating variable and identified six nodes, representing three AD-likelihood risk categories. According to the prevalence of AD observations within each node, RT_2_ classified nodes 1 and 2 as low-risk (in green), nodes 3 and 4 as middle-risk (in pink), and nodes 5 and 6 as high-risk (in red). Specifically, we found that node 6 (U-p53^2D3A8+^ ≥ 10.05) contained only AD patients, whereas in node 5 (9.35 ≤ U-p53^2D3A8+^ ≤ 10.05), the AD prevalence was 78%. When U-p53^2D3A8 +^ was less than 7.71, the risk of developing AD was very low, since most of the subjects in node 1 were stable-CN and stable-MCI (Figure S5). Considering the intermediate U-p53^2D3A8+^ threshold, the algorithm selected two well-known co-variables associated with AD risk: Mini-Mental State Examination (MMSE) and apolipoprotein E epsilon-4 (APOEε4). When MMSE ≥ 25.5 and APOEε4 were present, most of the subjects in node 3 were stable-MCI (37.50%) with a 25% risk of developing AD. In the absence of this AD genetic predisposing factor, the algorithm classified subjects in node 2 as lo-risk. Conversely, cognitive decline (MMSE < 25.5) combined with U-p53^2D3A8+^ levels between 7.71 and 9.35 placed individuals in node 4, in which 50% were represented by AD patients and 20% by AD converters (Figure 3a, Figure S5). 

Figure 3b summarizes results obtained by the algorithm in stratifying AD-likelihood risk of InveCe.Ab subjects (RT_2_, out of sample). Overall, RT correctly classified 86.67% of the cases in agreement with the clinical diagnosis. In detail, the algorithm classified 13 subjects as high-risk, among whom 11 (84.62%) developed AD within 4 years. It classified 65 low-risk subjects; among them, 62 (95.38%) were non-AD patients (CN or MCI). Twelve subjects were classified as medium-risk, where, in addition to plasma U-p53^2D3A8+^, APOEε4 presence and MMSE < 25.5 aided the classification. After 4 years, five developed AD while seven were stable MCI or progressed to MCI. The algorithm was superior in identifying those low-risk subjects (*p* < 0.001) compared to those at high-risk (*p* < 0.05) of developing AD, suggesting U-p53^2D3A8+^ could be a tool within the context of use (COU) of primary care settings. The value of U-p53^2D3A8+^ in contributing to the performance of the algorithm was demonstrated by excluding U-p53^2D3A8+^ from the RT analysis (Figure S6). 

Since the tumor suppressor p53, in all its multiple roles, is widely invoked in many conditions ranging from cancer to aging and age-related diseases [11,43,44], we report the results of plasma U-p53^2D3A8+^ tested in 114 elderly patients affected by different chronic diseases and multi-morbidities recruited in the population-based study ANZIANI IN-RETE [34]. In particular, subjects were stratified as a function of different disease types including cancer (20), cardiovascular (50), inflammatory (21), and metabolic (23) diseases (Table S8). In all these stratified groups, U-p53^2D3A8+^ values showed a similar trend to that found for Invece.Ab_stable CN and did not overlap with values found in InveCe.Ab_AD converters. (Figures S7 and S8, Table S9).

**Figure 4:**
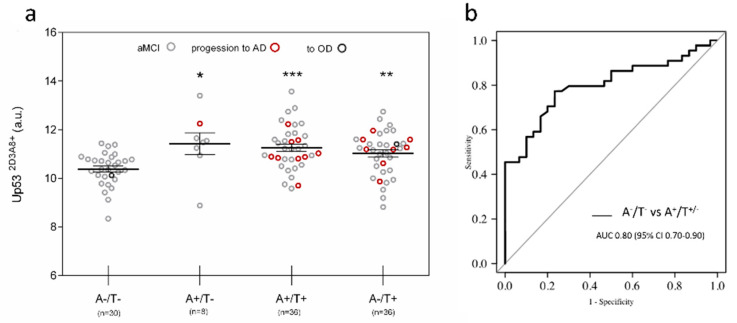
U-p53^2D3A8+^ in PharmaCog/E-ADNI subjects stratified according to CSF markers. Subjects were stratified using a multimodal classification scheme with CSF Aβ42 (A) and p-Tau (T) cutoff reported in the literature. (**a**) Scatter dot blots report the value of U-p53^2D3A8+^ in the different groups. The results obtained are represented as mean ± SEM. Red circle: aMCI to AD, dark gray circle: aMCI to other dementia. After a period of 6 to 30 months, 91 patients remained stable aMCI, apart from 1 who developed mixed AD + DLB dementia at 24 months (in the group A^−^/T^−^), 1 who progressed to DLB at 12 months (in the group A^−^/T^+^), and 18 who progressed to AD in a range of 6 to 30 months (9 in the A^+^/T^+^ group, 1 in the A^+^/T^-^ group, and 8 in the A^−^/T^+^ group). Wilcoxon test adjusted for multiple comparison: A^−^/T^−^ vs. A^+^/T^–^ (*p* < 0.05 *); A^+^/T^+^ (*p* < 0.001 ***) and A^-^/T^+^ (*p* < 0.01 **). One patient had missing CSF information. (**b**) ROC curve was used to distinguish A^−^/T^−^ versus those subjects with Aβ—positive status (A^+^/T^+/−^) with an AUC of 0.80. Additional information on the performance of U-p53^2D3A8+^ in discriminating these groups is reported in Figure S11. Results were extrapolated from a short-reference peptide standard curve and are expressed as arbitrary units (a.u).

U-p53^2D3A8+^ was also tested in the plasma samples of 111 aMCI derived from the PharmaCog/E-ADNI [26] longitudinal study, where CSF Aβ42, t-Tau, and p-Tau were considered. According to the measurement of CSF Aβ42 (A) and p-Tau (T), the A/T system (a multimodal classification scheme modified from Jack and colleagues [7]), 110 aMCI subjects were subdivided into four groups: A^−^/T^−^ (30), A^+^/T^−^ (8), A^+^/T^+^(36), and A^−^/T^+^ (36). U-p53^2D3A8+^ levels were found to be statistically higher in A^+^/T^+^ (*p* < 0.001), A^+^/T^−^ (*p* < 0.05), and A^−^/T^+^ (*p* < 0.01) compared to A^−^/T^−^ (Figure 4a) and showed an ROC with an AUC of 0.80 in discriminating A^−^/T^−^ versus A^−^/T^−/+^ (Figure 4b). 

**Figure 5:**
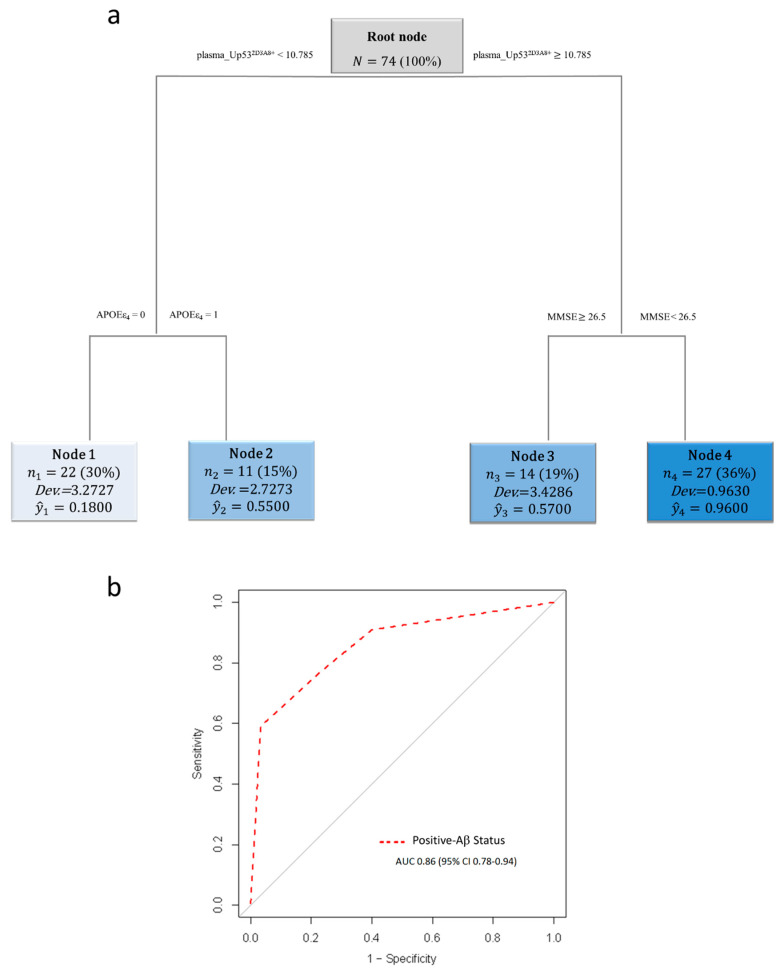
U-p53^2D3A8+^ plasma-based algorithm classification of Aβ status in PharmaCog/E-ADNI subjects. Schematic representation of the regression tree algorithm obtained by combining U-p53^2D3A8+^ with MMSE and APOEε4 to classify A^−^/T^−^ (*n* = 30) and Aβ^+^-subjects (A^+^/T^+/−^) (*n* = 44) (**a**). ROC curve showed RT diagnostic performance in discriminating patients with Aβ^+^ status. AUC = area under the curve (**b**). Cutoffs are expressed in arbitrary units (a.u.) and related only to the here-proposed in-house ELISA with a short-reference peptide used as internal standard.

Next, to investigate the ability of plasma U-p53^2D3A8+^ to identify Aβ^+^ subjects (A^+^/T^+/−^), we applied an RT algorithm combining U-p53^2D3A8+^ with MMSE and APOEε4. The resulting RT identified four distinct nodes, where plasma U-p53^2D3A8+^ was still the most important splitting variable that gave rise to two main branches (nodes 1 and 2 and nodes 3 and 4). According to a specific threshold of 10.79, 57%, and 96% of Aβ^+^ subjects branched into node 3 (U-p53^2D3A8+^ ≥ 10.79 and MMSE ≥ 26.5) and node 4 (U-p53^2D3A8+^ ≥ 10.79 and MMSE < 26.5), respectively. In particular, nine of those MCI in node 4 developed AD within 6–30 months. Of the aMCI in node 2 (U-p53^2D3A8+^ < 10.79 and no APOEε4), 55% were Aβ^+^ subjects who remained stable, apart from one who converted to AD. Node 1 (U-p53^2D3A8+^ < 10.78 and APOEε4) was represented by 82% of A^−^/T^−^ subjects (Figure 5a). The diagnostic performance of the algorithm in identifying Aβ-positive status showed an ROC with an AUC of 0.86 (95 CI%: 0.78–0.94) (Figure 5b). 

**Figure 6:**
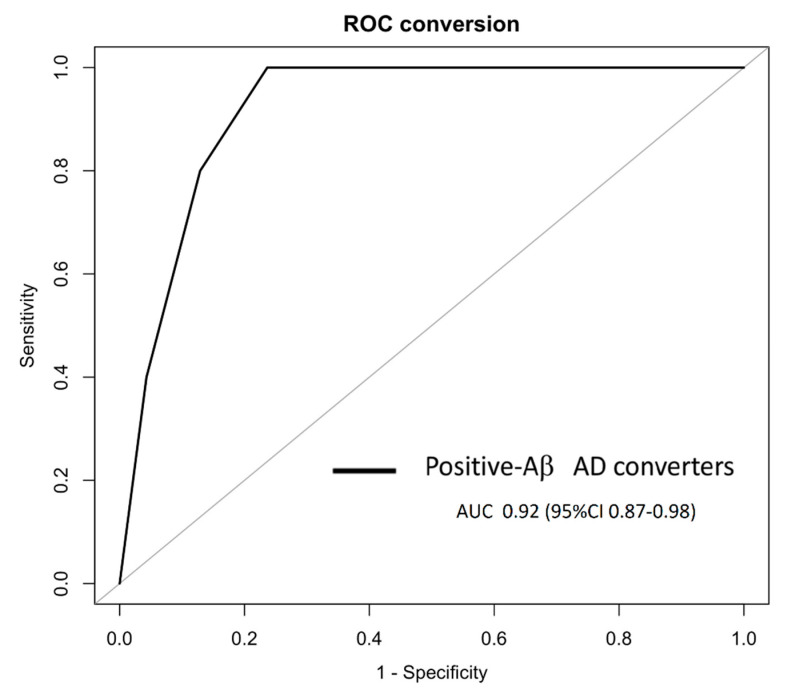
In PharmaCog/E-ADNI, the U-p53^2D3A8+^ plasma-based algorithm was able to identify at least 6–30 months prior to Aβ-positive AD conversion. The ROC derived from the regression tree algorithm was obtained using the PharmaCog/E-ADNI subset and combining U-p53^2D3A8+^ with MMSE and APOEε4 for classifying Aβ^+^-aMCI patients who will progress to AD after 6–30 months. Aβ^+^ AD converters: AUC 0.92 (95% CI: 0.87–0.98).

Among the 18 aMCI who converted to AD, 10 were classified according to the A/T system at baseline as Aβ^+^ (Table 1), and the U-p53^2D3A8+^ plasma-based algorithm showed a prognostic performance with an AUC of 0.92, a specificity of 78.50%, and a sensitivity of 100% in identifying Aβ^+^ aMCI who will develop AD after 6–30 months (Figure 6). 

## 4. Discussion

There is now general agreement that AD entails numerous neuropathologic changes associated with progression and neurodegeneration. In the current landscape of AD biomarkers that reflect the hallmarks of the disease, such as Aβ burden and pathological tau, there is an urgent need to identify additional markers of other pathological disease factors for inclusion in a biomarker panel for individually tailored precision AD diagnosis. U-p53^2D3A8+^ is a relatively unknown player within the setting of AD; nevertheless, it has the features of a credible marker of important biological processes. In our prior work, we identified the increased expression of an unfolded conformation of this transcription factor in fibroblast and blood cells derived from AD patients [45,46] and aMCI patients who converted to AD [47] as well as AD/MCI plasma specimens [41]. We confirmed the results in other biological frameworks, including immortalized lymphocytes derived from familial and sporadic AD patients [12].

In this study, we characterized and confirmed a new antibody (referred to as 2D3A8) as a reliable tool for recognizing an U-p53 conformational variant that is highly expressed in AD both at the preclinical and prodromal stages of the disease. In particular, plasma U-p53^2D3A8+^ was tested in a longitudinal study and then confirmed in a well-characterized cohort. U-p53^2D3A8+^ plasma levels correlated with the clinical evolution of the disease, as described by longitudinal analysis, and showed high accuracy in discriminating AD converters versus non-converters, as shown by ROC analyses. According to the Alzheimer’s Precision Medicine Initiative (APMI) working group [9], a blood-based biomarker of AD should provide a tool to assess subjects that are subjectively cognitively normal or with very early signs of cognitive decline in the primary care setting, allowing identification of the at-AD-risk subset who require further evaluation. In this context, the prognostic value of U-p53^2D3A8+^, derived by an integrated RT algorithm based on the rolling window procedure, might meet this need. Overall, 86.67% of U-p53^2D3A8+^ node assignments agreed with clinical diagnosis (77.48–92.62%). This method allowed the automated stratification of CN individuals and MCI at different degrees of AD risk. Plasma U-p53^2D3A8+^ was found more relevant for identifying low-AD-risk subjects, providing a tool to be used in the COU in primary care settings.

U-p53^2D3A8+^ validation will require cohorts well-characterized in term of APOε4 status, CSF, and/or PET scan Aß [7,9]. As misdiagnosis rates could exceed 20% [48], we also tested plasma U-p53^2D3A8+^ in PharmaCog/E-ADNI samples. The aMCI of the PharmaCog/E-ADNI study, stratified according to the A/T multimodal classification scheme, highlighted a good correspondence between the high levels of U-p53^2D3A8+^ and AD-associated brain pathology (Figure 4). In addition, the RT based on U-p53^2D3A8+^ combined with APOEε4 genotype and MMSE further showed robust performance in distinguishing aMCI classified as A^+^/T^+/−^ versus A^−^/T^−^ (AUC of 0.86), suggesting U-p53^2D3A8+^ in plasma reflects what is occurring in the brain. The algorithm was also able to show high prognostic performance in identifying Aβ^+^-AD converters (AUC of 0.92). Correspondingly, U-p53^2D3A8+^ levels could be complementary to other AD biomarkers, such as CSF or plasma Aß [10,49] and therefore could be included in a panel of plasma biomarkers, supporting a personalized AD diagnosis. 

Machine learning has been of interest in the biomarkers research of complex diseases as a platform that can harvest information from biochemical and clinical sources into an integrated system [50]. Here, we trained different RTs whose differences in terms of thresholds were justified by the different outcomes used in the analysis (i.e., AD vs. non-AD; A^−^/T^−^ vs. A^+^/T^−/+^). The algorithm was able to highlight the relevance of U-p53^2D3A8+^ in AD since, in all the datasets used, plasma U-p53^2D3A8+^ is the first splitting variable requested by the algorithm to describe AD using statistical language.

It is noteworthy that the application of machine learning to early detection and automated classification of Alzheimer’s disease has recently gained considerable attention because it allows the integration of big and deep biomedical data. The ability to deal with “big data science” accompanied by the implementation of integrative disease is a crucial starting step within a precision medicine strategy [9]. On the other hand, it is important to be aware of the problem of overfitting that occurs when the learning algorithm describes the random error or noise instead of the underlying data relationship. For this reason, in this study, Bootstrap Method and cross-validated prediction have been applied to strengthen the robustness of data obtained. 

The evidence of U-p53^2D3A8+^ expression in disease progression warrants further investigation of its role in AD pathogenesis. From a mechanistic point of view, a possible link between the p53 conformational variant and Aβ has been suggested by investigating HIPK2–p53 signaling in different in vitro studies [21,51]. When Aβ is present at nanomolar levels, throughout the inhibition of HIPK2, it induces the expression of metallothionein 2A, which, endowed with Zn-chelating activity, sequesters metals from the DNA-binding-domain of p53 and induces its conformational changes, which in turn inhibits its activity [21]. In addition, a low-grade pro-oxidant environment, instead activating p53 intracellular pathways, affects its tertiary structure, inducing conformational changes and the loss of its activity [13,52,53]. Since p53 regulates a heterogeneous repertoire of biological functions [43,44], including neuronal outgrowth and neuronal connectivity protection [14,21], regulation of innate immunity [54], and redox homeostasis [21,55,56], we hypothesize that the expression of this conformational variant in the early stage of the disease might contribute to synapse dysfunction, inflammation, and oxidative stress. Therefore, p53-misfolding variants might represent a signature of early AD pathological events, including Aß accumulation, and redox imbalance and immune activation, leading eventually to oxidative stress and chronic inflammation, respectively. The precision medicine paradigm for AD biomarker discovery has highlighted the importance of assay development. 

## Figures and Tables

**Table 1:** Demographic and clinical profile of the subjects. Demographic and clinical variables and genotype frequency of the APOEε4 polymorphisms of all the subjects. N: number; M: male; F: female; MMSE: Mini-Mental State Examination; CN: cognitively normal subjects; MCI: mild cognitive impairment; aMCI: amnestic mild cognitive impairment; AD: Alzheimer’s disease; CSF: cerebrospinal fluid. Data are expressed as mean ± standard deviation (SD). For female prevalence and genotype, absolute frequencies (%) are reported.

	InveCe.Ab		PharmaCog/E-ADNI
No. plasma samples	264 *		111
Classification	CN	MCI	aMCI
N. subjects	64	26	111 ^a^
Sex: female, n (%)	37 (57.81%)	8(30.77%)	64 (57.66%)
Age, mean (SD)	73 (1.22)	73.28 (1.36)	69.12 (7.55)
APOEε4 status, n (%)	14 (21.87%)	8 (30.77%)	52 (46.85%)
Level of education (SD) ^b^	1.14 (0.65)	1.16 (0.54)	1.37 (0.50)
MMSE (SD)	27.56 (2.28)	26.5 (2.11)	26.41 (1.83)
Conversion to AD, N	10/64	9/26	18/111
Conversion time (min–max), months	24–48	24–48	6–30
AD-related biomarkers			
CSF Aβ42 level (SD), pg/mL			676.41 (290.72)
CSF p-Tau level (SD), pg/mL			70.31 (36.98)
CSF t-Tau level (SD), pg/mL			503.82 (373.44)

* Baseline, T_1_, and T_2_ plasma samples. Missing plasma samples at T_1_ (2 Stable MCI and 1 MCI-to-AD) and at T_2_ (3 MCI-to-AD already converted at T_1_); ^a^ Missing CSF (1); ^b^ Level of education was assigned as follows: illiterate (0), from 3 to 12 years (1), from 13 to 18 years (2), and more than 18 years (3).

## Data Availability

Data are available upon request to giulia.abate@unibs.it or marika.vezzoli@unibs.it

## Supplementary Materials

The following are available online at https://www.mdpi.com/2075-4426/11/1/14/s1, Table S1: Inter- and Intra-variability of different batches of antibody comparing both negative and positive internal Quality Controls (QCs). Figure S1: 2D3A8 antibody recognizes an open variant of p53 recombinant protein. Figure S2: Blocking-epitope peptide inhibits 2D3A8 binding to p53 recombinant protein. Figure S3: Reproducibility of InveCe.Ab CN/CN to AD data from an FDA-certified independent laboratory. Table S2: InveCe.Ab population study: Description and conversion rate within the follow-up. Table S3: InveCe.Ab_Neuropsicological test battery (NTB) at baseline. Table S4: InveCe.Ab neuropsychological test battery at different time points. Table S5: Descriptive statistics of U-p53 ^2D3A8+^ in InveCe.Ab cohort across follow-up. Table S6: Two separated models’ descriptions used in LME. Figure S4: Detailed description of RTs applied to InveCe.Ab dataset where the rolling window procedure is used for evaluating Out-Of-Sample model performances. Table S7: Performances of RTs reported in Figure 3 in-sample (ten-fold Cross-Validation) and Out-Of-Sample. Figure S5: U-p53 ^2D3A8+^ stratified with respect to the partition (Nodes) identified by RT_2_. Figure S6: ROCs obtained by the RT models with or without U-p53^2D3A8+^ on InveCe.Ab dataset. Table S8: Demographic and clinical description of 114 subjects derived from “ANZIANI IN RETE” recruitment used in this study. Figure S7: Boxplots on U-p53^2D3A8+^ for the different disease categories in “ANZIANI IN RETE” recruitment. Table S9: Kruskal–Wallis test computed on U-p53^2D3A8+^ stratified respect different subgroups of diseases in “ANZIANI IN RETE”. Figure S8: Naive Bayes Plot for U-p53^2D3A8+^ for the different disease categories.
