# Supplementary material for: A Conformation Variant of p53 Combined with Machine Learning Identifies Alzheimer Disease in Preclinical and Prodromal Stages
Source: J Pers Med. 2020 Dec 26;11(1):14. doi: 10.3390/jpm11010014 (PMC7823360; doi:10.3390/jpm11010014)

Supplementary material

2D3A8 Antibody production and characterization: additional description of the method

The overall study was carried on with two different batches of 2D3A8 antibodies (IgM). The first batch derived from hybridoma-cultured medium, produced and purified at University of Brescia.
Batch#2 was purified from hybridoma-cultured medium by a GMP, ISO-13485 manufacturer.

In details, different batches were used as follow:

Batch	Plasma Samples Analyzed	Operator
Batch#1	- InveCe.Ab<br>- Anziani in rete cohort	UNIBS
Batch#2	- PharmaCog plasma	UNIBS

The two batches performances on negative and positive QCs (quality controls), used in our in-house ELISA assay are reported in ***Supplementary Table 1***.
According with the ICH and FDA guidelines, (CV% intra-assay <10% and inter-assay <15%), the two batches showed acceptable intra- and inter- variability assay.

		INTER-VARIABILITY		INTRA-VARIABILITY	
		Negative QCs CV%	*Positive QCs CV%*	*Negative QCs CV%*	*Positive QCs CV%*
Batch#1	*UNIBS*	*7.87%*	*13.4 %*	*3.57 %*	*7.99%*
Batch#2	*Hycult Biotech*	*12.3%*	*4.07%*	*3.6%*	*2.9%*

Supplementary Table 1. Inter- and Intra-variability of different batches of antibody comparing both negative and positive internal Quality Controls (QCs).

2D3A8 Antibody specificity and reproducibility: additional description of the method

2D3A8 antibody recognizes an open variant of p53 recombinant protein.
2D3A8 antibody recognizes an open variant of p53**.** In detail, 2D3A8 antibody recognizes a linear epitope (aa 282-297) between the DNA binding domain and the conjunction region with the tetramerization domain. This epitope is less accessible when the protein is in wild type conformation and become exposed following conformational changes. As reported in Supplementary figure 1, 2D3A8 antibody preferentially recognized EDTA-treated recombinant p53. EDTA is a zinc-chelating agent that subtracting Zinc atom induces p53 conformational changes towards a misfolding isoform. The specificity of the 2D3A8 antibody is highlighted by the linearity of the signal, that is strongly higher when p53 is conformationally open.

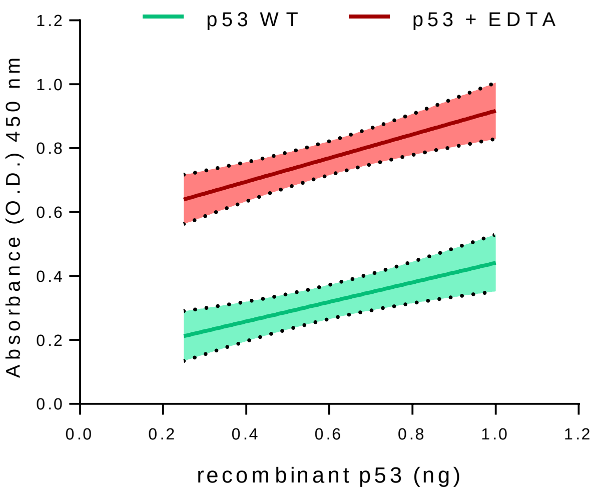

Supplementary figure 1. Recombinant p53 protein produced in baculovirus (ActiveMotif) was exposed to EDTA, that subtracting Zn atom gets lost p53 wild type conformation towards a misfolding phenotype. The graph reports results of the immunoassay performed with 2D3A8 antibody on different amounts of recombinant p53 (0.3-0.5-1ng) before and after EDTA treatment. Data are expressed as Optical Density (O.D.).

Blocking-epitope peptide inhibits 2D3A8 binding to p53 recombinant protein

To demonstrate 2D3A8 is specific to the interaction with p53 protein we blocked 2D3A8 antibody through competition with a peptide that matches the sequence of 2D3A8-epitope.

Thus, 1 µg of 2D3A8 antibody has been pre-incubated with increasing doses of the blocking peptide (ratio Ab: blocking peptide: 1:1; 1:2; 1:3 and 1:4) and then used in the in-house ELISA assay. 5 ng of p53 recombinant protein treated with EDTA has been tested. We found that increasing doses of blocking epitope peptide are able to inhibit the binding of 2D3A8 antibody with p53 protein thus supporting 2D3A8 specificity.

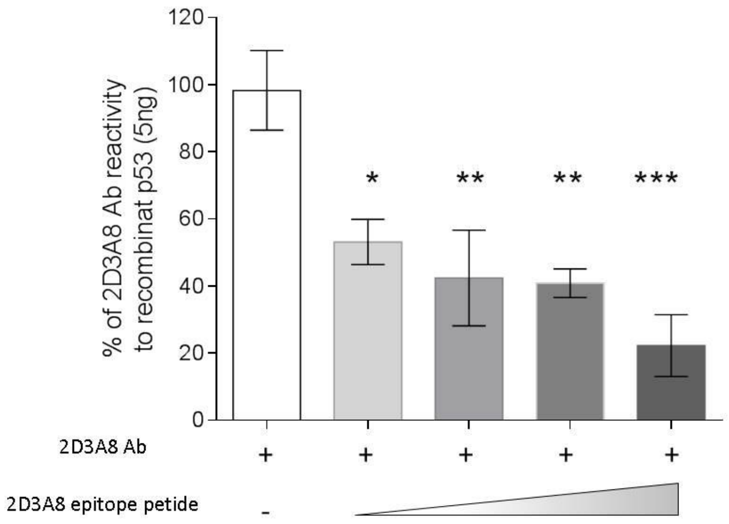

Supplementary figure 2. 5 ng of recombinant p53 + EDTA has been incubated with 2D3A8 antibody both in presence or absence of increasing doses of 2D3A8 blocking epitope-peptide. Epitope peptide has been pre incubated according with the following antibody/blocking peptide ratio: 1:1 (light grey) $p<0.05$; 1:2 (medium grey) $p<0.01$; 1:3 (medium dark grey) $p<0.01$ and 1:4 (dark grey) $p<0.001$. No blocking peptide pre-incubation is reported with the white bar. Data are expressed as mean ± s.e.m.

Reproducibility of 2D3A8 in-house ELISA test performed by an independent laboratory.
The *bona fide* of the antibody performance is demonstrated by the reproducibility of the data obtained by Future Diagnostic Inc. (FD), a company specialized for (IVD) assay development that is also an ISO13485:2016 and FDA registered manufacturing facility in the Netherland. Part of InveCe.Ab samples were re-tested in blind by FD lab. In particular 138 samples (stable CN and CN converted to AD) were re-tested with 2D3A8 antibody batch#2. Data obtained have been analyzed by an independent statistician. FD results showed a good diagnostic accuracy (AUC=0.80) with a sensitivity of **0.90** and specificity of **0.72** in distinguish stable CN vs CN converted to AD. Figure S3, shows the reproducibility of the results

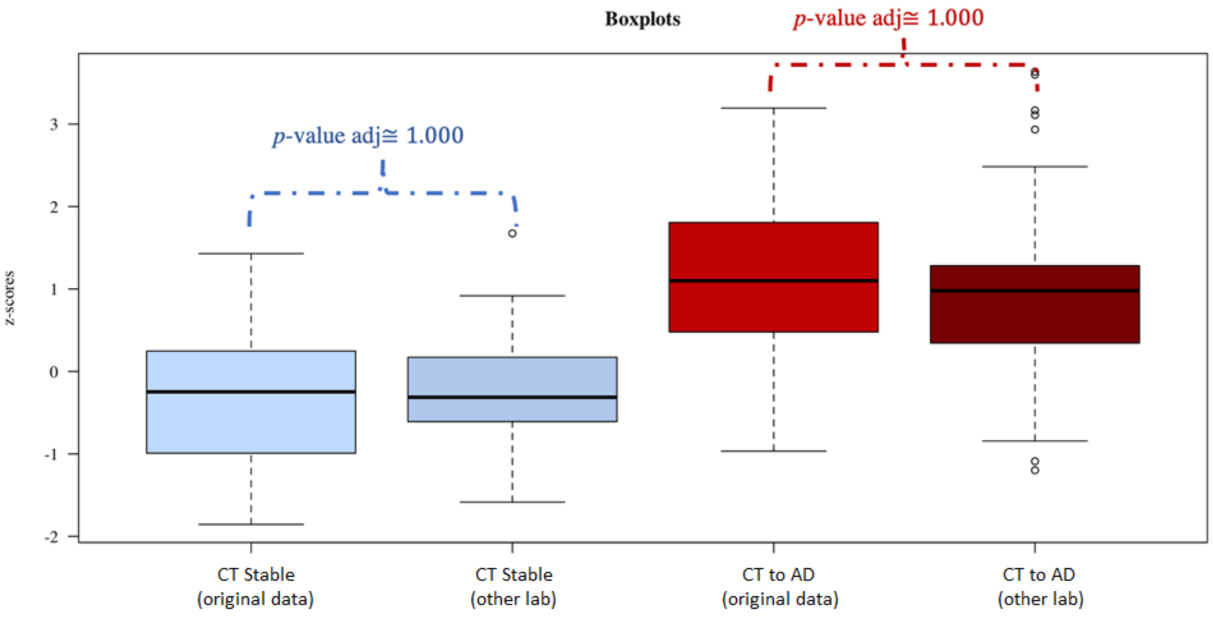

Supplementary figure3: ***Reproducibility of InveCe.Ab CN/CN to AD data from an FDA certified independent laboratory.*** *Z-* Score Boxplot. stratified for diagnosis. Z score is computed in order to overcome the scale problems. Moreover, Kruskall-Wallis p-values (adjusting for multiple comparison) were computed.

Statistical analysis: additional description of the method

Linear Mixed Effects Model

Two separate Linear Mixed Effect (LME) models have been performed: one comparing stable CN with CN to AD, and the other comparing stable MCI with MCI to AD. Models were fit through restricted maximum likelihood (REML), also known as residual maximum likelihood. Standard likelihood method (ML) was not adopted since it is reported to be biased in small samples. Assumptions about normality of residuals and homoscedasticity were checked through visual inspection. The description of the model output is described here below.

Level 1

$$\mathrm{U-p53^{2D3A8+}} = \pi_{0i} + \pi_{1i} time_{ij} + \varepsilon_{ij}$$

Level 2

$$\pi_{0i} = \gamma_{00} + \gamma_{01} time + \tau_{0i}$$

$$\pi_{1i} = \gamma_{10} + \gamma_{11} time + \tau_{1i}$$

The level 1 submodel represents the individual change in plasma_U-p53^{2D3A8+} occurring over time (years). The plasma_U-p53^{2D3A8+} at occasion j, for person I, is a function of an intercept, which corresponds to participant's plasma_U-p53^{2D3A8+} level at baseline π_{0i}, and one slope parameter. A linear term is included to capture the rate of change over time π_{1i}. Level-1 residuals represent the portion of subject i's value of plasma_U-p53^{2D3A8+} levels at time j not predicted by the model.

The level 2 submodels capture systematic interindividual differences in trajectories. At level 2, the level 1 intercept and slope parameters become the outcomes, and they are predicted as a function of four parameters: γ_{00}, γ_{10},$\gamma_{10}$and γ_{11}. These parameters are the average intercept (γ_{00}), the hypothesized difference in the average true initial status between diagnosis groups (γ_{01}), the average true annual rate of change (γ_{10}), the effect of diagnostic faith in the average true monthly rate of change (γ_{11}). Each submodel has its own residual (τ_{0i},τ_{1i}) that permits the level-1 parameters of one person to differ stochastically from those of the others. Of note: time was treated as unstructured by using patient's actual age at each assessment. To have a better interpretability of $\gamma_{00}$and γ_{01} parameters, baseline was set at 70 years old.

Participants and clinical phenotyping: Supplementary Tables

Supplementary Table 2. InveCe.Ab population study: Description and conversion rate within the follow up.

The table reported the 1039 Cognitively Normal (CN) 4 years follow up conversion rate (CN to AD=0.96 %; CN to OD =1.15%; CN to MCI=10.49%; Stable CN=66.6%); and the 101 MCI 4 years follow up conversion rate (MCI to AD=9.9 %; MCI to OD =15.84%; MCI Stable=39.60%).
Not all the participants attended to the following visits (T1 and T2) after the baseline and are depicted in the following table as "drop out".

Baseline_2010	T_1_ 2012	T_2_ 2014
	803 CN	639 CN<br>82 MCI<br>3 AD<br>6 OD<br>67 drop out
	4 AD<br>4 OD	4 AD<br>4 OD
	72 MCI	29 CN<br>23 MCI<br>2 AD<br>1 OD<br>17 drop out
	156 drop out	24 CN<br>4 MCI<br>1 AD<br>1 OD<br>126 drop out

Baseline_2010	T_1_ *2012*	T_2_ *2014*
**101 MCI**	17 CN	10 CN<br>6 MCI<br>1 drop out
	4 AD<br>7 OD	4 AD<br>7 OD
	54 MCI	5 AD<br>9 OD<br>32 MCI<br>8 drop out
	19 drop out	1 AD<br>2 CN<br>2 MCI<br>14 drop out

Supplementary Table 3. InveCe.Ab _Neuropsicological test battery (NTB) at Baseline.

All participants from InveCe.Ab dataset have been run through Neuropsychological assessments addressing several cognitive areas using the applicable instruments, as listed below (**Table S3**). The different subgroups have been then selected according with age, gender, comorbidity index*, severity index* and clinical category matched. Global cognition was assessed using MMSE [1], corrected for age and years of education following the normative data published by Magni et al.[2] Verbal episodic memory was evaluated using the revised version of the Babcock Story Recall Test and the Rey Auditory-Verbal Learning Test[3] . Language was assessed using the Phonemic and Semantic Verbal Fluency Test[4] . Executive functions were gauged using Raven's Coloured Matrices[5] and Clock Drawing Test[6] . Simple and divided attention, and attention control were tested using the Attention Matrices and Trail Making Test[7] . Finally, visuospatial skills were evaluated using the Rey-Osterrieth Complex Figure (copy and recall) [8]. Each evaluation session was preceded by an informal interview to evaluate potential interfering factors and to help the participants feel at ease. The medical evaluation together with the neuropsychological assessment provided information useful to calculate the prevalence of dementia and cognitive impairment. In **Table S4** NTB have been divided according with each subgroups (Stable CN, Stable MCI, CN to MCI, MCI to AD, CN to AD) for each time points (Baseline, T_1 and T_2) during the follow up study.

Cognitive Processes	Neuropsichological Test Battery (NTB)	CN	MCI	*p* value
	N. of subjects	64	26	
	Mean severity index (SD)*	1.43 (0.267)	1.53 (0.198)	0.07
	Mean comorbidity index (SD)*	1.72 (1.43)	2.33 (1.41)	0.06
	Geriatric_Depression_Scale	1.75 (2.65)	2.00 (2.09)	0.7
**Executive functions**	Raven's Progressive Matrices	28.95 (4.29)	22.89 (5.07)	<0.001
	Clock drawing test	18.45 (1.96)	17.59 (2.26)	0.06
**Language**	Verbal Fluency (FIVeCa)	17.64 (3.48)	14.57 (2.34)	<0.001
**Verbal episodic memory**	Rey Auditory Verbal Learning Test (PaReyI)	40.02 (9.68)	32.8 (6.58)	0.001
	Rey Auditory Verbal Learning Test differite (PaReyD)	8.22 (3.04)	4.83 (2.61)	<0.001
	Memory test (Babcock story recall)	11.86 (2.16)	8.27 (3.48)	<0.001
**Visual spatial skill**	Rey–Osterrieth Complex Figure Drawing Test_Recall	16.14 (4.26)	10.58 (3.59)	<0.001
	Rey–Osterrieth Complex Figure Drawing Test_Copy	29.96 (5.97)	26.13 (6.81)	0.02
**Attention**	Trial Making form A	30.79 (21.51)	34.93 (18.32)	0.39
	Trial Making form B	69.93 (61.82)	141.67 (89.03)	<0.001
	Trial Making form B-A	50.38 (53.01)	106.34 (77.3)	0.003
	Attentional Matrix	48.96 (7.24)	42.06 (6.24)	<0.001

Supplementary Table 4. **InveCe.Ab neuropsychological test battery at different time points.**

Cognitive Processes	Neuropsichological Test Battery (NTB)	Stable CN				CN to AD				MCI to AD				CN to MCI				stable MCI			
		T0	24 m	48m	p value	T0	24 m	48m	p value	T0	24 m	48m	p value	T0	24 m	48m	p value	T0	24 m	48m	p value
	Geriatric_Depression_Scale	1.00 (1.09)	1.18 (1.23)	1.05 (1.32)	0,84	2.90 (2.88)	5 (3.06)	5.38 (4.90)	0,07	2.60 (2.17)	3.88 (3.04)	3 (3.65)	0,83	2.82 (4.23)	2.18 (2.96)	3.71 (4.47)	0,07	1.65 (2.03)	1 (2.07)	1 (1.54)	0,25
	MMSE	27.66 (2.28)	27.95 (1.63)	27.61 (1.82)	0,81	26.43 (1.46)	23.81 (1.27)	17.12 (4.94)	**0,001**	24.59 (1.64)	22.89 (3.77)	18.59 (3.88)	**<0.001**	26.9 (1.4)	26.41 (2.12)	25.41 (2.09)	**0,02**	24.41 (1.94)	26 (2.06)	25.27 (2.55)	0,08
**Executive functions**	Raven's Progressive Matrices	30.42 (3.89)	34.64 (3.80)	31.88 (3.90)	0,00	25.39 (4.15)	25.60 (2.48)	20.75 (1.50)	0,28	23.50 (6.65)	26.17 (6.11)	26.58 (4.97)	**0,02**	27.42 (3.84)	29.82 (2.95)	28.97 (2.78)	**0,03**	22.53 (4.08)	29.07 (3.95)	27.09 (3.20)	0,00
	Clock drawing test	18.74 (1.35)	18.71 (1.04)	17.95 (2.03)	0,92	16.60 (3.60)	14.29 (2.69)	10.44 (3.71)	**0,005**	17.30 (2.45)	15.22 (5.54)	15 (2.94)	0,76	19 (1.17)	17.47 (2.50)	17.29 (1.36)	**<0.0001**	17.76 (2.19)	17.20 (1.97)	16.71 (2.23)	0,08
**Language**	Verbal Fluency (FIVeCa)	18.58 (3.01)	18.69 (2.93)	19.11 (3.12)	0,25	14.87 (2.75)	13.08 (1.63)	11.50 (3.25)	0,05	13.94 (2.42)	11.75 (2.47)	8.71 (3.41)	0,06	17.19 (4.01)	17.33 (4.11)	15.69 (5)	**0,05**	14.91 (2.30)	15.75 (2.65)	14.32 (1.99)	0,31
**Verbal episodic memory**	Rey Auditory Verbal Learning Test (PaReyI)	42.84 (9.73)	40.56 (8.15)	41.73 (7.14)	0,43	32.16 (4.44)	22.82 (5.57)	23.30 (3.84)	**0,05**	35.24 (4.11)	28.78 (5.07)	24.67 (5.25)	**0,03**	37.29 (8.86)	34.05 (6.40)	31.12 (7.33)	**0,01**	31.81 (7.22)	32.56 (5.24)	27.88 (4.44)	0,05
	Rey Auditory Verbal Learning Test differite (PaReyD)	9.39 (2.85)	8.60 (2.45)	9.13 (2.27)	0,39	5.03 (2.77)	2 (2.77)	2.05 (2.90)	0,50	4.51 (2.76)	3.12 (2.86)	2.05 (2.90)	0,52	7.04 (2.03)	5.65 (1.74)	5.87 (1.68)	**0,02**	4.95 (2.62)	5.90 (2.42)	5.92 (1.66)	0,52
	Memory test (Babcock story recall)	12.62 (2.02)	12.60 (2.68)	13.17 (2.33)	0,07	11.44 (1.49)	5.74 (4.49)	3.78 (4.74)	**0,03**	8.59 (2.85)	8.48 (3.99)	8.58 (3.93)	0,60	10.4 (2.06)	9.73 (3.93)	11.55 (2.13)	0,22	8.09 (3.87)	9.27 (3.96)	9.43 (3.69)	0,35
**Visual spatial skill**	Rey–Osterrieth Complex Figure Drawing Test_Recall	17.13 (4.17)	17.74 (5.17)	17.37 (5.87)	0,94	14.21 (4.97)	4.13 (5.83)	N.A.	0,34	12.46 (4.46)	12.19 (3.08)	9.25 (1.77)	0,06	14.97 (3.87)	11.36 (3.03)	12.20 (3.65)	**0,02**	9.77 (2.93)	8.70 (4.21)	10.28 (2.26)	0,50
	Rey–Osterrieth Complex Figure Drawing Test_Copy	31.33 (4.61)	31.13 (3.82)	31.43 (4.30)	0,30	27.50 (9.18)	22.50 (2.83)	N.A.	0,60	28.64 (7.85)	27.31 (8.19)	29.17 (7.31)	0,17	28.22 (6.60)	24.13 (4.82)	25.67 (5.28)	0,48	25.03 (6.26)	24.78 (3.51)	26.81 (5.39)	0,97
**Attention**	Trial Making form A	22 (17.09)	19.68 (14.27)	22.03 (15.50)	0,58	47 (27.96)	77.71 (54.58)	91.29 (75.80)	0,19	33.44 (19.16)	36.83 (18.21)	62.17 (35.90)	0,13	40.29 (17.61)	33.65 (16.88)	52.13 (36.17)	0,36	35.71 (18.40)	39.92 (23.98)	49.35 (35.49)	0,14
	Trial Making form B	57.77 (59.72)	44.23 (48.42)	54.92 (44.27)	0,99	48 (38.15)	149 (137.18)	N.A.	0,36	129.71 (111.57)	113.67 (77.51)	N.A.	0,86	98.43 (66.2)	93.82 (48.95)	141.30 (75.74)	0,14	149.27 (76.44)	144.60 (90.91)	57.60 (37)	0,21
	Trial Making form B-A	43 (51.22)	29.39 (44.19)	50.83 (34.43	0,69	40 (22.07)	89 (76.37)	N.A.	0,36	97 (90.24)	77 (64.09)	N.A.	0,83	63 (60.39)	61.36 (33.71)	97.10 (59.02)	0,11	112.27 (71.91)	115.80 (79.07)	36 (27.87)	0,24
	Attentional Matrix	51.53 (5.78)	48.89 (5.28)	50.46 (6.41)	0,65	43.34 (40.25)	31.21 (9.39)	37.90 (6.27)	**0,04**	41.03 (5.65)	41.17 (7.16)	39.15 (10.51)	0,53	45.83 (6.03)	42.52 (7.36)	40.82 (7.02)	**0,00**	42.60 (6.63)	40.92 (8.47)	39.98 (7.21)	0,13

References

1. Folstein. M. F.. Folstein. S. E. & McHugh. P. R. 'Mini-mental state'. A practical method for grading the cognitive state of patients for the clinician. *J. Psychiatr. Res.* **12**. 189–198 (1975).
2. Magni. E.. Binetti. G.. Bianchetti. A.. Rozzini. R. & Trabucchi. M. Mini-Mental State Examination: a normative study in Italian elderly population. *Eur. J. Neurol.* **3**. 198–202 (1996).
3. Carlesimo. G. A.. Sabbadini. M.. Fadda. L. & Caltagirone. C. Different components in word-list forgetting of pure amnesics. degenerative demented and healthy subjects. *Cortex.* **31**. 735–745 (1995).
4. Carlesimo. G. A.. Caltagirone. C. & Gainotti. G. The Mental Deterioration Battery: normative data. diagnostic reliability and qualitative analyses of cognitive impairment. The Group for the Standardization of the Mental Deterioration Battery. *Eur. Neurol.* **36**. 378–384 (1996).
5. Basso. A.. Capitani. E. & Laiacona. M. Raven's coloured progressive matrices: normative values on 305 adult normal controls. *Funct. Neurol.* **2**. 189–194 (1987).
6. Mendez. M. F.. Ala. T. & Underwood. K. L. Development of scoring criteria for the clock drawing task in Alzheimer's disease. *J. Am. Geriatr. Soc.* **40**. 1095–1099 (1992).
7. Giovagnoli. A. R. *et al.* Trail making test: normative values from 287 normal adult controls. *Ital. J. Neurol. Sci.* **17**. 305–309 (1996).
8. Caffarra. P.. Vezzadini. G.. Dieci. F.. Zonato. F. & Venneri. A. Rey-Osterrieth complex figure: normative values in an Italian population sample. *Neurol. Sci. Off. J. Ital. Neurol. Soc. Ital. Soc. Clin. Neurophysiol.* **22**. 443–447 (2002).

Supplementary Tables and Figures

Supplementary Table 5. Descriptive statistics of U-p53 $^{2D3A8+}$ in InveCe.Ab cohort across follow-up .
*N**in appendix= *N* converted to AD at T_1.

U-p53 $^{2D3A8+}$	stable CN (37)	CN to MCI (17)	CN to AD (10)^{5*}	stable MCI (17)	MCI to AD (9)^{4*}	*p value adjusted*
**Baseline**						
Mean ± SD	6.33 ± 1.03	6.60 ± 1.50	7.53 ± 1.37	6.44 ± 1.08	7.31 ± 1.75	0.72[a)]
Median	6.47	6.91	8.11	6.79	6.73	***0.03***[b)]
Min-Max	4.22 – 8.18	4.24 – 8.64	5.53 – 9.07	4.37 – 8.15	5.73 – 10.87	0.72[c)]
T_1						
Mean ± SD	6.64 ± 1.13	7.03 ± 0.98	8.39 ± 1.39	7.16 ± 1.13	8.10 ± 1.98	0.31[a)]
Median	6.85	7.13	8.71	7.21	8.56	***0.004***[b)]
Min-Max	4.92 – 8.79	5.02 – 8.91	5.46 – 10.03	5.36 – 9.16	4.27 – 10.07	0.28[c)]
T_2						
Mean ± SD	6.26 ± 1.00	7.71 ± 1.02	9.15 ± 1.17	7.49 ± 1.38	9.68 ± 1.29	***<0.0001***[a)]
Median	6.32	7.73	9.04	7.35	9.73	***<0.0001***[b)]
Min-Max	4.29 – 8.04	5.58 – 9.47	7.47 – 11.25	5.56 – 10.85	7.68 – 11.11	***0.003***[c)]

Wilcoxon rank sum test computed on:
a) stable CN vs CN to MCI
b) stable CN vs CN to AD
c) stable MCI vs MCI to AD
p values are adjusted for multiple comparisons

Supplementary Table 6. Two separated model's descriptions used in LME. One comparing stable CN with CN converting to AD (**a**). and the other comparing stable MCI with MCI converting to AD (**b**). 95% CI are reported .

Model output

Fixed Effects

Initial status (baseline)

- **Intercept:** the average value of plasma_U-p53^{2D3A8+} level at baseline for stable CN (arbitrarily selected at 70 years of age. to simplify interpretability of coefficients).
- **Diagnosis group**: the estimated average difference of plasma_U-p53^{2D3A8+} level for CN that will convert to AD vs stable CN at baseline.

Rate of change

- **Age**: the estimated average individual yearly rate of change of plasma_U-p53^{2D3A8+} level
- **Diagnosis group:** difference in population average annual rate of change between CN converter to AD and stable CN

Random Effects (Variance components)

- **Level-1 (within-person).** $\boldsymbol{\varepsilon_{ij}}$. summarizes the net (vertical) scatter of the observed data around individual i's hypothesized change trajectory
- **Level-2 (In initial status).** $\boldsymbol{\tau_{0i}}$. it's the population residual variance of true initial status. controlling for diagnosis group
- **Level-2 (In rate of change).** $\boldsymbol{\tau_{1i}}$. it's the population residual variance of true rate of change. controlling for diagnosis group

a) CN-to-AD vs CN

Fixed Effects	**Estimate**	**Std. Err.**	**p-value**
Initial status			
Intercept. γ_{00}	6.40	0.27	<0.001
Diagnosis group (CNtoAD vs stable CN). γ_{01}	-0.08	0.61	0.89
Rate of change			
Age. γ_{10}	0.03	0.04	0.37
Diagnosis group (CNtoAD vs stable CN). γ_{11}	0.33	0.09	<0.001
Random Effects	**Estimate**	**Std. Err.**	**95% CI**
Level-1 (within-person). ε_{ij}	0.57	0.14	0.35-0.90
Level-2 (In initial status). τ_{0i}	1.04	0.74	0.25-4.24
Level-2 (In rate of change). τ_{1i}	0.01	0.02	0.01-0.01

b) MCI-to-AD vs MCI

Fixed Effects	**Estimate**	**Std. Err.**	**p-value**
Initial status			
Intercept. γ_{00}	5.79	0.46	<0.001
Diagnosis group (MCItoAD vs stable MCI) γ_{01}	-0.47	0.77	0.54
Rate of change			
Age	0.24	0.09	0.01
Diagnosis group (MCItoAD vs stable MCI) γ_{11}	0.33	0.15	0.03
Random Effects	**Estimate**	**Std. Err.**	**95% CI**
Level-1 (within-person). ε_{ij}	1.19	0.26	0.78-1.82
Level-2 (In initial status). τ_{0i}	0.05	0.22	0.01-346.47
Level-2 (In rate of change). τ_{1i}	0.02	0.03	0.01-0.29

Regression Trees Performance

Supplementary Figure 4. Detailed description of RTs applied to InveCe.Ab dataset where the *rolling window* procedure is used for evaluating Out-Of-Sample model performances of the models obtained. The algorithm has grown two trees using the variables in both cases (grey box). It was calibrated on two different training set: (*i*) Baseline (yellow box) and (*ii*) Baseline +T_1 (orange box). At each step, the model is tested on fresh data: (i) T_1 (red box) and (ii) T_2 (white box) respectively. The two models obtained are very similar with respect to variables and thresholds selected, providing the stability of the predictors. Consequently, authors selected the more accurate RT_2 while RT_1 is available upon request.

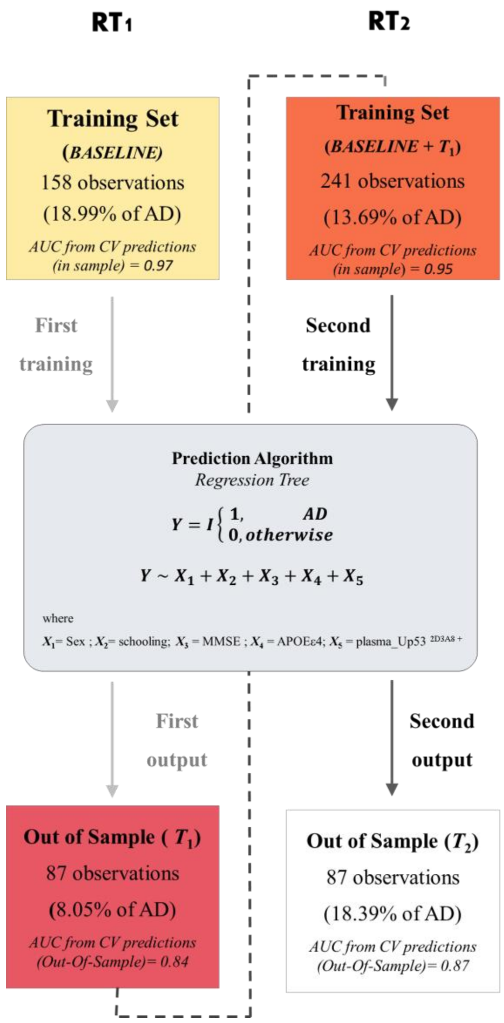

		Observation (N.)	AUC	Specificity	Sensitivity
RT1	Training set 1 *Cross-validated in-sample performance*	158	0.97	0.91	0.97
RT1	Test set 1 *Out-of sample performance*	87	0.84	0.75	0.86
RT2	Training set 2 *Cross-validated in-sample performance*	241[a]	0.95	0.89	0.94
RT2	Test set 2 *Out-of sample performance*	87[b]	0.87	0.92	0.81

[a] For predicting T_2, we excluded from the training set the 4 subjects already converted in T_1
[b] 87 observations in test set 2 correspond to plasma samples available for T_2 , but to note among 4 MCI to AD already converted in T_1, 3 of them do not have T_2 plasma sampling

Supplementary Table 7. Performances of RTs reported in figure 3 in-sample (ten-fold Cross-Validation) and Out-Of-Sample.

Supplementary Figure 5. U-p53 $^{2D3A8+}$ stratified respect the partition (Nodes) identified by RT_2

RT_2 induce a partition of patients in 6 different nodes with different risk level (in green low risk, in dark red high risk) of conversion to AD. Their U-p53^{2D3A8+} values have been stratified respect to this Nodes. We reported: i) the composition of each node respect to the clinical classification where *n* (%) is the number (and %) of observations in the training set (baseline + T_1 =241 values). and ii) the composition of each node respect to the conversion in T_1 and, prospectively, in T_2.

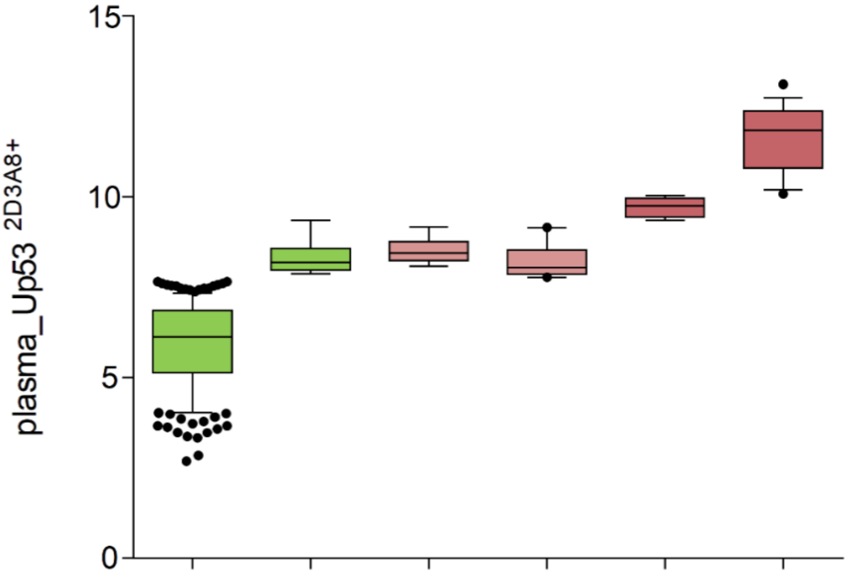

	Node 1	Node 2	Node 3	Node 4	Node 5	Node 6
**Number of values**	176	19	8	10	9	19
**Mean. Plasma_Up53^{2D3A8+} (SD)**	5.88 (1.20)	8.27 (0.40)	8.52 (0.35)	8.21 (0.49)	9.70 (0.27)	11.57 (0.94)
Composition of each node respect the clinical classification n (%) Training set: Baseline + T_1						
CN	106 (60.23%)	11 (57.89%)	3 (37.50%)	2 (20%)	0	0
MCI	70 (39.77%)	8 (42.11%)	3 (37.50%)	3 (30%)	2 (22.22%)	0
AD	0	0	2 (25%)	5 (50%)	7 (77.78%)	19 (100.00%)
Composition of each node respect the conversion in T_1 and, perspectively, in T_2 n (%) Training set: Baseline + T_1						
Stable CN	93 (52.84%)	7 (36.84%)	2 (25.00%)	0	0	0
CN to MCI	25 (14.20%)	7 (36.84%)	1 (12.50%)	1 (10.00%)	0	0
CN to AD in T_1	0	0	0	0	0	0
CN to AD in T_2	2 (1.14%)	1 (5.26%)	0	2 (20.00%)	1 (11.11%)	0
Stable MCI	54 (30.68%)	2 (10.53%)	3 (37.50%)	2 (20.00%)	0	0
MCI to AD in T_1	0	0	0	0	2 (22.22%)	1 (5.26%)
MCI to AD in T_2	2 (1.14%)	2 (10.53%)	0	0	1 (11.11%)	0
AD	0	0	2 (25.00%)	5 (50.00%)	5 (55.55%)	18 (94.74%)

Supplementary Figure 6. ROCs obtained by the RT models with or without U-p53^{2D3A8+} on InveCe.Ab dataset. In order to understand the importance of U-p53^{2D3A8+} in RT_2 (which is trained on baseline+T_1), the Cross Validated (CV) predictions with (black line) and without U-p53^{2D3A8+} (dashed blue line) were computed and compared in a unique graph. RT where U-p53^{2D3A8+} is excluded, shows an AUC of 0.77 (specificity = 0.81 and sensibility = 0.67). The De Long test for the comparison of the AUCs confirms that RT_2 with U-p53^{2D3A8+} (AUC =0.95) is significantly different from the nested model where U-p53^{2D3A8+} is excluded (p-value<0.001). Repeating the same analysis also Out-Of-Sample we confirmed the same results (data shown upon request).

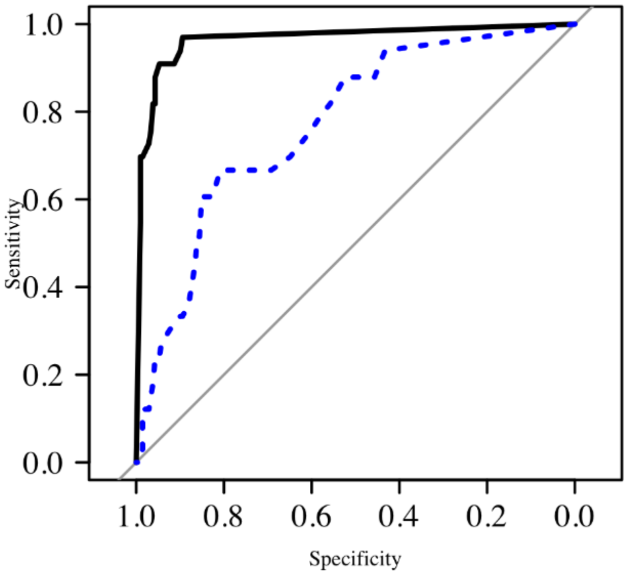

Supplementary Table 8: Demographic and clinical description of 114 subjects derived from "ANZIANI IN RETE" recruitment used in this study.

	Cancer	Cardiovascular Disease	Inflammatory Disease	Metabolic Disease	*p*_value
N. of Plasma samples	20	50	21	23	
Sex: females. n.(%)	10 (50%)	19 (38.0%)	6 (28.6%)	9 (39.1%)	0.57
Age. mean (SD)	79.45 (6.37)	81.74 (7.07)	82.71 (7.98)	83.09 (9.36)	0.42
MMSE (SD)	27.35 (3.10)	27.85 (2.38)	27.79 (2.44)	28.134 (2.25)	0.79
Level of education (SD)	1.57 (0.51)	1.31 (0.47)	1.67 (0.49)	1.67 (0.49)	0.13
U-p53 $^{2D3A8+}$ (SD)	5.59 (0.99)	5.78 (1.46)	6.10 (0.94)	6.22 (1.43)	0.34

Supplementary Figure 7. Boxplots on U-p53^{2D3A8+} for the different disease categories in "ANZIANI IN RETE" recruitment.

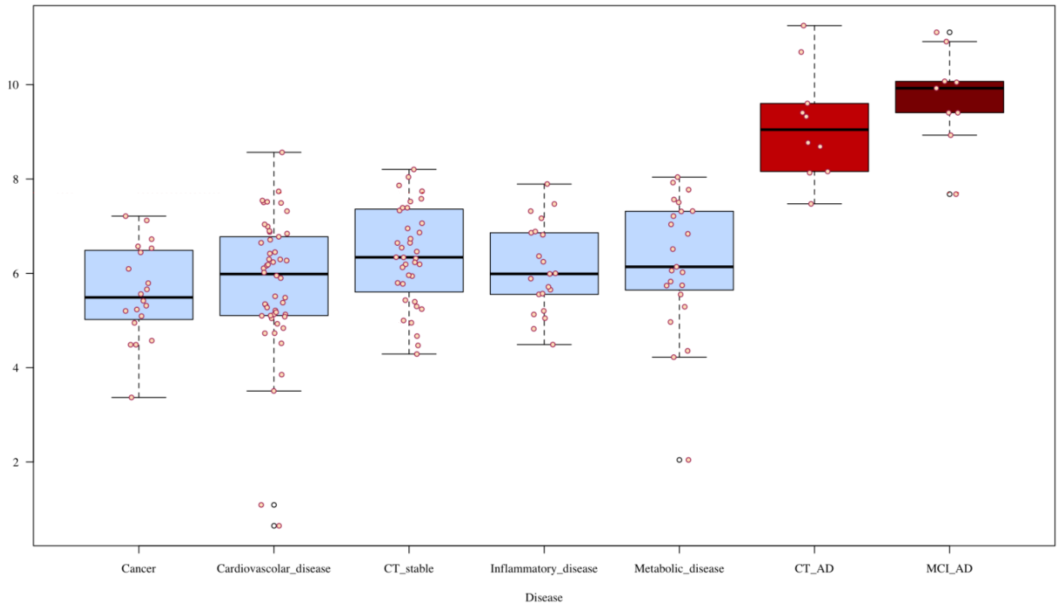

Supplementary Table 9: Kruskal-Wallis test computed on U-p53^{2D3A8+} stratified respect different subgroups of diseases (p-values are adjusted for multiple comparisons)

	**Adjusted *p*-values**
Cancer vs Cardiovascular disease	1.00
Cancer vs CT stable	0.13
Cancer vs Inflammatory disease	1.00
Cancer vs Metabolic disease	0.46
Cancer vs CT to AD	***<0.001***
Cancer vs MCI to AD	***<0.001***
Cardiovascular disease vs CT stable	0.46
Cardiovascular disease vs Inflammatory disease	1.00
Cardiovascular disease vs Metabolic disease	1.00
Cardiovascular disease vs CT to AD	***<0.0001***
Cardiovascular disease vs MCI_AD	***<0.0001***
CT stable vs Inflammatory disease	1.00
CT stable vs Metabolic disease	1.00
CT stable vs CT to AD	***<0.0001***
CT stable vs MCI to AD	***<0.0001***
Inflammatory disease vs Metabolic disease	1.00
Inflammatory disease vs CT to AD	***<0.0001***
Inflammatory disease vs MCI to AD	***<0.0001***
Metabolic disease vs CT to AD	***<0.0001***
Metabolic disease vs MCI to AD	***<0.0001***
CT to AD vs MCI to AD	1.00

p-values <0.05 are highlighted in bold and italic

Supplementary Figure 8. Naive Bayes Plot for U-p53^{2D3A8+} for the different disease categories.

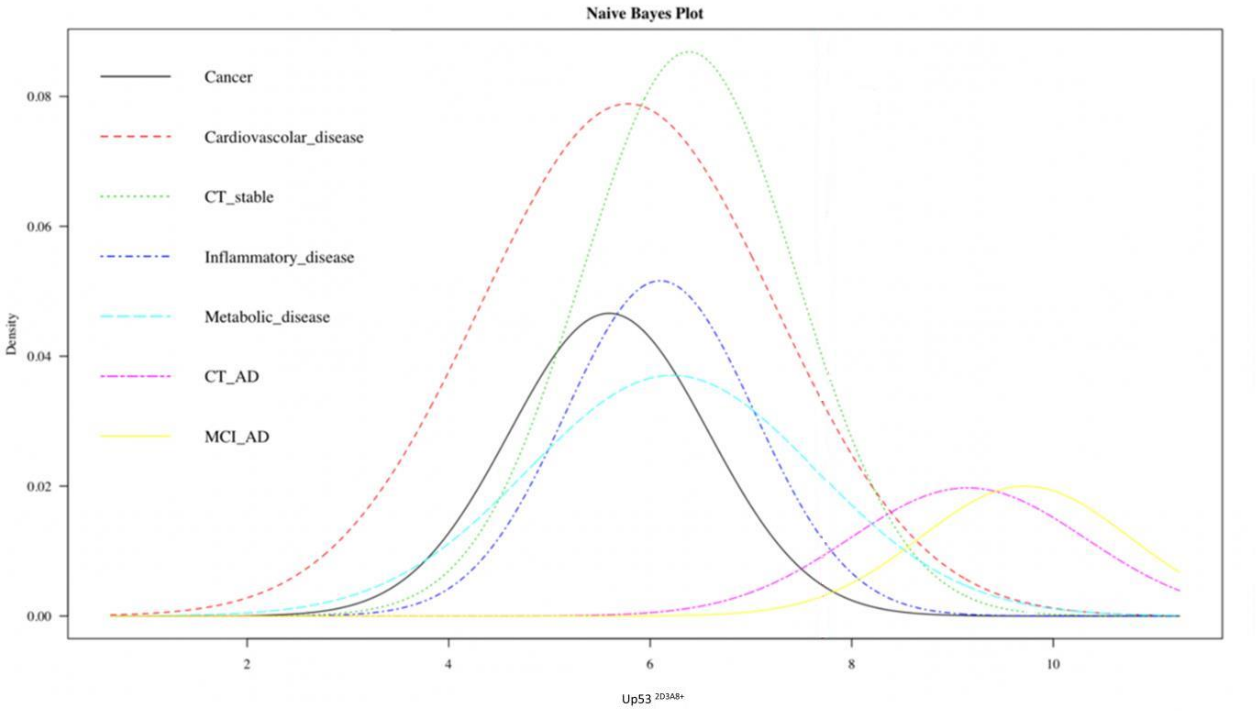

Supplement: Supplementary file 1 [file jpm-11-00014-s001.pdf]
